# Remimazolam-Based Anesthetic Management in a Patient with Severe Aortic Stenosis and Myelodysplastic Syndrome-Related Thrombocytopenia: A Case Report

**DOI:** 10.3390/jcm14238371

**Published:** 2025-11-25

**Authors:** Sou-Hyun Lee, Seung Bae Cho, Hyojun Choo, Jongone Park, Sung-Hye Byun

**Affiliations:** 1 Department of Anesthesiology and Pain Medicine, School of Medicine, Kyungpook National University, Kyungpook National University Hospital, Daegu 41944, Republic of Korea; youlion6@gmail.com; 2 Department of Anesthesiology and Pain Medicine, Kyungpook National University Chilgok Hospital, Daegu 41404, Republic of Korea; chosbboy@naver.com; 3 Department of Anesthesiology and Pain Medicine, School of Medicine, Kyungpook National University, Kyungpook National University Chilgok Hospital, Daegu 41404, Republic of Korea; junaace@naver.com (H.C.); permutation2@naver.com (J.P.)

**Keywords:** aortic valve stenosis, case report, myelodysplastic syndromes, pancytopenia, remimazolam

## Abstract

**Background/Objectives:** Severe aortic stenosis (AS) poses major anesthetic challenges because cardiac output is highly dependent on preload and heart rate, and abrupt afterload reduction or tachycardia may precipitate ischemia and cardiovascular collapse. Coexisting myelodysplastic syndrome (MDS) with severe thrombocytopenia further increases the perioperative bleeding risk, which we considered particularly important in the anesthetic planning for this patient. We report a case of laparoscopic anterior resection in a patient with severe AS and thrombocytopenia, highlighting a transfusion strategy adjusted according to the patient’s response and remimazolam-based anesthesia. To the best of our knowledge, there have been no previous reports describing remimazolam-based total intravenous anesthesia achieving stable hemodynamics without vasopressor support in a patient with severe AS and MDS–related thrombocytopenia. **Method:** A 78-year-old man with previously diagnosed MDS and chronic pancytopenia, whose baseline platelet counts (PLTs) ranged from 20,000 to 40,000/μL, was found to have severe AS (aortic valve area, 0.73 cm^2^; mean pressure gradient, 42 mmHg) during preoperative evaluation for laparoscopic anterior resection. After platelet transfusions titrated to his response, the patient’s PLT increased to 93,000/μL before surgery. Anesthesia was induced and maintained with remimazolam and remifentanil, which were chosen to prevent afterload reduction associated with propofol. **Results:** Hemodynamics, including arterial pressure, cardiac index, systemic vascular resistance index, and cerebral oxygen saturation, remained stable without vasopressor support. Transient systolic hypertension during surgical stimulation was controlled using remifentanil titration and esmolol. Recovery and the postoperative course were uneventful, and the patient was discharged in a stable condition. **Conclusions:** Remimazolam-based total intravenous anesthesia can provide hemodynamic stability without vasopressors in high-risk patients with severe AS, and a transfusion strategy adjusted step by step according to the patient’s response can be effective for optimizing PLTs while minimizing the transfusion-related risks of MDS-associated thrombocytopenia.

## 1. Introduction

Severe aortic stenosis (AS) is characterized by fixed narrowing of the left ventricular outflow tract. Consequently, cardiac output is highly dependent on the heart rate (HR) and preload. These patients are vulnerable to myocardial ischemia due to preexisting high afterload and concentric left ventricular (LV) hypertrophy. In these patients, general anesthesia and surgery are associated with high perioperative risks. Typical problems include reduced coronary perfusion due to diastolic hypotension and tachycardia, and hemodynamic instability secondary to abrupt changes in afterload [1,2,3]. Maintaining sinus rhythm, preventing tachycardia, and adequately preserving diastolic blood pressure (DBP) and mean arterial pressure (MAP) are central to anesthetic management, as coronary perfusion occurs primarily during diastole [3,4].

Myelodysplastic syndrome (MDS) is characterized by pancytopenia secondary to ineffective hematopoiesis [5]. Severe thrombocytopenia significantly increases the risk of perioperative bleeding, necessitating adequate platelet transfusion to maintain hemostasis and preload stability, particularly in patients with severe AS. However, repeated transfusions can lead to human leukocyte antigen (HLA) alloimmunization, whereas MDS itself is associated with hypersplenism and chronic inflammation. These factors can collectively contribute to platelet transfusion refractoriness [6], making it difficult to achieve the desired platelet increment. This creates a therapeutic dilemma. Insufficient transfusion may exacerbate bleeding and compromise preload. In contrast, excessive transfusion may precipitate pulmonary edema or transfusion-associated circulatory overload (TACO), for which severe AS itself is a predisposing factor [7,8]. In addition, transfusion-related acute lung injury (TRALI) can be catastrophic in this context [7]. Therefore, achieving an appropriate balance between ensuring hemostatic adequacy and preventing volume overload requires a transfusion strategy that is tailored to the patient’s condition and adjusted according to the clinical response.

Propofol may cause abrupt afterload reduction and diastolic hypotension secondary to vasodilation [9,10]. Because of these hemodynamic concerns, we selected remimazolam as the anesthetic agent in this case. Remimazolam is an ultra-short-acting benzodiazepine that has been reported to provide relatively stable hemodynamics and to be suitable for induction and maintenance in patients with severe AS [1,11,12,13].

This report describes the case of a 78-year-old patient with severe AS and marked thrombocytopenia secondary to MDS who successfully underwent laparoscopic anterior resection. We highlight (1) perioperative optimization using platelet transfusion adjusted according to the patient’s response to achieve hemostatic balance and (2) hemodynamic management using remimazolam–remifentanil total intravenous anesthesia and multimodal monitoring, which enabled stable hemodynamics without vasopressor use. To our knowledge, no previous report has described remimazolam-based total intravenous anesthesia in a patient with concurrent severe AS and MDS–related thrombocytopenia requiring perioperative platelet optimization. This case adds practice-relevant information. It illustrates the feasibility and key clinical considerations of remimazolam-based anesthesia and transfusion management tailored to the patient’s response in the setting of coexisting high-risk cardiac and hematologic disorders.

## 2. Case Presentation

A 78-year-old man (height, 156.4 cm; weight, 54.4 kg; BMI, 22.25 kg/m^2^) was scheduled to undergo laparoscopic anterior resection for sigmoid colon cancer. He had been diagnosed with MDS (RAEB-1, IPSS intermediate) seven years earlier, indicating a moderate hematologic risk [14]. He had been receiving monthly hypomethylating agent therapy, and his disease remained stable, although chronic pancytopenia persisted. Complete blood counts performed during the two months preceding surgery showed persistently severe thrombocytopenia (platelet count [PLT], 20,000–40,000/μL), consistent with his known MDS-related pancytopenia.

His cardiovascular history included angina, diagnosed 10 years earlier, which was managed with sublingual nitroglycerin as needed. During preoperative evaluation for the planned colorectal surgery, severe AS that had not been previously assessed was incidentally found. On physical examination, his vital signs were stable, lung auscultation was clear without crackles, and no peripheral edema was present. Cardiac auscultation demonstrated a regular rhythm with a systolic murmur, consistent with severe AS. Chest computed tomography angiography revealed significant atherosclerotic stenosis of the mid-left anterior descending and proximal right coronary arteries and moderate-to-severe AS. Transthoracic echocardiography performed at the time of anemia-related dyspnea revealed preserved LV systolic function (ejection fraction, 58%), without regional wall motion abnormalities or evidence of heart failure. Echocardiographic parameters were consistent with severe AS (Table 1). He had no history of cardiac or valvular interventions. The cardiothoracic team considered valve replacement surgery contraindicated because of the patient’s severe thrombocytopenia and high bleeding risk.

On 22 August, while awaiting surgery, the patient developed transient dyspnea corresponding to New York Heart Association (NYHA) III, associated with anemia (hemoglobin concentration [Hb] 5.0 g/dL). The cardiology team administered two units of filtered red blood cells (RBCs) along with diuretics, resulting in symptomatic improvement. This episode was attributed to anemia-related decompensation rather than AS progression.

The hematology team recommended perioperative platelet transfusion and prophylactic antibiotics, aiming for Hb ≥10 g/dL and PLT ≥ 100,000/μL at the time of surgery. Although current guidelines recommend platelet transfusion when the PLT is <50 × 10^3^/μL for adult patients undergoing major non-neuraxial surgery, a more conservative target was chosen in this case. We were concerned that even a brief episode of hypovolemia could precipitate hemodynamic instability in the setting of severe AS.

He was elderly but lived with family members who provided adequate support. Despite his age, he maintained a stable general condition and psychosocial environment, with no factors expected to interfere with perioperative management.

**Figure 1:**
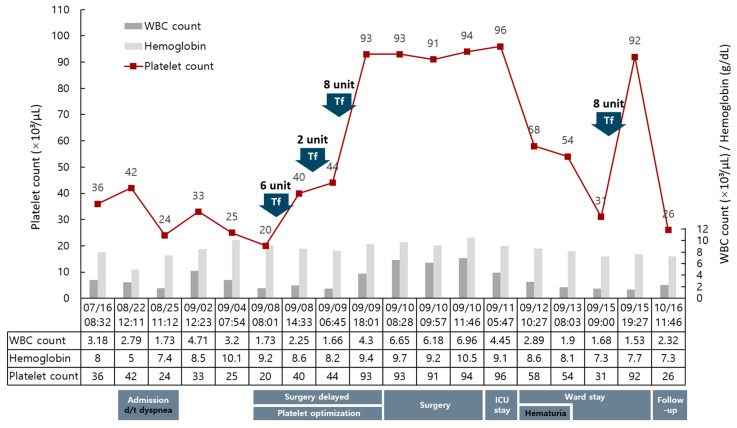
Perioperative trends of complete blood count and major clinical events in a patient with myelodysplastic syndrome–related thrombocytopenia. The red line indicates platelet count (×10^3^/μL). Arrows indicate transfusion (Tf) events and the corresponding units. The gray and light gray bars represent the white blood cell (WBC) count (×10^3^/μL) and hemoglobin concentration (g/dL), respectively. The platelet transfusions administered on 8 and 9 September effectively increased the platelet counts to the perioperative target. The lower panel summarizes the perioperative timeline, including admission for dyspnea, preoperative platelet optimization, surgery, ICU and ward stays, transient hematuria, and outpatient follow-up.

On 4 September, the day before the scheduled surgery, the operation was postponed for further evaluation of the transverse colon lesion. At that time, the PLT was 25,000/μL. In accordance with the hematology team’s recommendation, platelet transfusions were initiated cautiously to reach the target level while minimizing the risk of hypovolemia in severe AS. Six units of random donor platelets (RDPs) were transfused on 8 September, and the PLT increased from 20,000 to 40,000/μL after 6 h. Two additional units were transfused in the same evening, and by the following morning, the PLT had increased to 44,000/μL. Although the post-transfusion increment was not fully sufficient, it indicated that refractoriness was unlikely according to the corrected count increment (CCI) [6]. The surgery was rescheduled on 10 September, based on the adequacy of the response. Twelve units of RDPs and one unit of apheresis platelets (equivalent to six RDP units) were secured, but further supply was uncertain. Based on serial CCI assessment, 8 units of platelets were transfused on 9 September, and the PLT had increased to 93,000/μL by 18:00. Further transfusion was withheld, and 10 units were reserved on the day of surgery. The Hb was corrected from 8.2 to 9.4 g/dL after transfusion of 1 unit of packed RBCs (Figure 1).

Standard monitoring, including electrocardiography, pulse oximetry, and non-invasive blood pressure, was applied on the day of surgery. Prophylactic antibiotics (cefazedone and metronidazole) were administered intravenously before surgical incision in accordance with the institutional protocol. Radial arterial cannulation was performed under ultrasound guidance while the patient was awake to enable continuous blood pressure monitoring. The anesthetic goals were to prevent bradycardia, maintain adequate preload, and prevent abrupt afterload reduction. Propofol administration was avoided due to the risk of vasodilation-induced diastolic hypotension. Anesthesia was induced and maintained using remimazolam and remifentanil. Remimazolam was started at 6 mg/kg/h, and remifentanil was administered by target-controlled infusion with an effect-site concentration (Ce) of 2 ng/mL, based on the Minto pharmacokinetic model. Rocuronium (50 mg) was administered after loss of consciousness, and tracheal intubation was performed using a McGrath videolaryngoscope. Anesthesia was maintained with remimazolam 1–2 mg/kg/h. The pre-induction blood pressure and HR were 168/45 mmHg (MAP, 86 mmHg) and 72 bpm, respectively. Intraoperatively, efforts were made to maintain MAP, DBP, and HR within target ranges of 70–80 mmHg, ≥60 mmHg, and 50–80 bpm, respectively. A systolic blood pressure (SBP) of up to 170 mmHg was permitted, as in our experience, maintaining a slightly higher systolic pressure is often necessary to preserve diastolic perfusion in patients with severe AS. Phenylephrine was prepared as the first-line treatment for hypotension, and norepinephrine bolus/infusion planned if additional support was required. The central venous catheter was inserted under ultrasound guidance, and the cardiac index, stroke volume variation, and systemic vascular resistance index (SVRI) were monitored continuously. The depth of anesthesia was monitored using the patient state index (PSI), which was maintained between 30 and 50 throughout the surgery, indicating an adequate hypnotic depth. Cerebral oximetry was also performed, and the regional oxygen saturation remained within 20% of the baseline values, suggesting stable cerebral perfusion.

**Figure 2:**
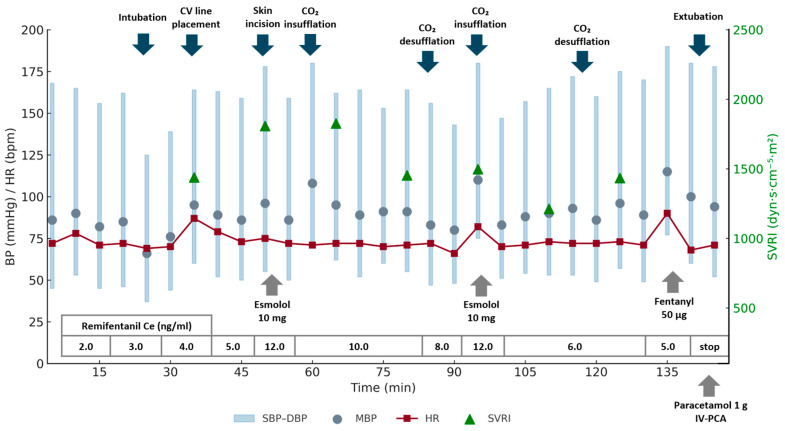
Intraoperative hemodynamic monitoring during laparoscopic anterior resection under remimazolam–remifentanil anesthesia. Systolic and diastolic blood pressures (SBP–DBP range; blue bars), mean arterial pressure (MAP; gray circles), heart rate (HR; red line), and systemic vascular resistance index (SVRI; green triangles) are shown. The lower table indicates the effect-site concentration (Ce) of remifentanil at each time point. The arrows denote key intraoperative events (intubation, central venous line placement, skin incision, CO_2_ insufflation/desufflation, and extubation), as well as the timing of drug administration (esmolol, fentanyl, paracetamol, and IV-PCA). MAP and DBP were preserved throughout anesthesia, and hemodynamic stability was maintained, despite transient fluctuations in SVRI.

The peri-induction laboratory tests confirmed a PLT of 93,000/μL and an Hb of 9.7 g/dL. During surgery, transient decreases in the calculated SVRI were observed. At each time point when the SVRI was low, the patient’s MAP remained stable overall, although the DBP intermittently fell below the target level. Since the SBP was already elevated at 160–170 mmHg, vasopressor administration was not required. Hemodynamic surges during skin incision and CO_2_ insufflation were managed by increasing the Ce of remifentanil to 12 ng/mL and administering esmolol 10 mg twice (Figure 2). The complete blood count results during surgery confirmed a PLT of 91,000/μL and an Hb of 9.2 g/dL. Intraoperative transfusion was not required. The surgery proceeded without complications. To provide analgesia and facilitate smooth emergence, fentanyl (50 μg) and acetaminophen (1 g) were administered near the end of the surgery. Remimazolam infusion was maintained until the completion of suturing and dressing, after which it was discontinued. For complete reversal of neuromuscular blockade and restoration of full consciousness to ensure airway patency and adequate spontaneous ventilation, sugammadex (200 mg) and flumazenil (0.2 mg) were administered intravenously. The patient regained full consciousness within 1 min and was safely extubated.

Recovery in the post-anesthesia care unit (PACU) was stable, without re-sedation. The patient was alert and oriented upon emergence from anesthesia and remained hemodynamically stable. His SBP was allowed to reach 170 mmHg, and intermittent doses of labetalol (5 mg) were administered twice as needed.

Postoperative analgesia was provided via an intravenous patient-controlled analgesia (IV-PCA) device containing fentanyl 1200 μg and ramosetron 0.6 mg, diluted with normal saline to a total volume of 60 mL. The PCA was programmed to deliver a background infusion of 0.5 mL/h (10 μg/h of fentanyl) and a patient-controlled bolus dose of 0.5 mL (10 μg) with a 15 min lockout interval. In the PACU, the patient reported a pain score of 6 on the numerical rating scale (NRS), and additional analgesia consisting of 50 μg of intravenous fentanyl and 30 mg of ketorolac was administered. The NRS score subsequently decreased to 4 before discharge from the PACU. During the first 6 h after surgery, the NRS remained around 5, and two doses of intravenous tramadol 50 mg were administered. Thereafter, the NRS score decreased to 2, and pethidine was administered as needed.

The patient was observed in the surgical intensive care unit (ICU) for 24 h postoperatively due to his comorbidities. Throughout the PACU and ICU stay, he remained alert and oriented, consistent with an estimated RASS (Richmond Agitation–Sedation Scale) score of 0. No clinical signs of delirium were identified. He was transferred to the ward on postoperative day 1, reported satisfactory pain control, and expressed overall satisfaction with the PCA regimen.

While recovering in the ward, a minor event occurred: the Foley catheter was inadvertently self-removed, resulting in transient pinkish hematuria without clot retention. The urine cleared spontaneously within a day, and cystoscopy or bladder irrigation was not required. Urology recommended maintaining the Foley catheter for two weeks and performing peri-catheter retrograde urethrography thereafter. The Hb and PLT gradually declined from 8.6 g/dL and 58,000/μL, measured earlier after surgery, to 7.3 g/dL and 31,000/μL, respectively. These values were comparable to the patient’s preoperative and follow-up baseline levels, suggesting that the decrease reflected his underlying hematologic condition rather than blood loss attributable to hematuria. Considering his severe AS and chronic thrombocytopenia, 1 unit of RBCs and 8 units of platelets were administered as conservative measures to maintain hemodynamic and hematologic stability.

The patient remained asymptomatic and was discharged the following day. At the one-month outpatient follow-up, his laboratory findings remained near his chronic baseline without bleeding events. The surgical site healed well without evidence of infection or anastomotic complications, and he reported no symptoms related to AS.

The Institutional Review Board of Kyungpook National University Chilgok Hospital in Daegu, Republic of Korea, granted exemption from ethical approval for this case (KNUCH 2025-10-036) because the clinical data were de-identified. The patient provided informed consent for the publication of this case report.

## 3. Discussion

This report describes the successful management of a patient with severe AS and profound thrombocytopenia secondary to MDS who underwent major abdominal surgery. Each condition independently increases the perioperative risk. Consequently, their coexistence presents a significant challenge for anesthesiologists and necessitates simultaneous hemodynamic stabilization and bleeding control.

The fixed obstruction of the outflow tract in severe AS renders the cardiac output highly dependent on the preload and HR. Bradycardia and hypovolemia should be prevented. Chronically elevated afterload leads to LV hypertrophy, which increases myocardial oxygen demand and predisposes the patient to ischemia. Coronary perfusion occurs during diastole; therefore, sufficient diastolic pressure and duration are crucial. This makes tachycardia and sudden afterload reduction particularly hazardous [2,3,4]. Valve intervention (aortic valve replacement or transcatheter aortic valve implantation) is recommended for patients with symptomatic disease [15,16]. However, thrombocytopenia precluded surgical and transcatheter interventions in this case.

Thrombocytopenia increases the risk of perioperative bleeding, which may reduce preload, provoke sympathetic activation, and worsen tachycardia-induced coronary malperfusion. The hypertrophied LV, with its high oxygen demand and impaired diastolic compliance, further limits compensatory mechanisms and exacerbates them [3,4]. Pancytopenia secondary to MDS results from ineffective hematopoiesis driven by abnormal stem cells [5]. Although hypomethylating agents promote differentiation, they primarily delay the progression and transformation to acute myeloid leukemia rather than restore normal hematopoiesis. Transfusion refractoriness is common, even when the marrow response improves platelet production, and is associated with repeated transfusions, HLA alloimmunization, hypersplenism, and chronic inflammation, resulting in reduced platelet survival [6].

Given these hematologic and cardiovascular vulnerabilities, large-volume platelet transfusions should be avoided. Excessive volume loading may precipitate pulmonary edema in severe AS [8,17], and TRALI can be catastrophic. Severe AS itself has been reported as a risk factor for TACO [7], warranting extreme caution during transfusion. To minimize the risk of TACO and TRALI, a restrictive transfusion strategy should be implemented [18]. This approach entails rigorously assessing the transfusion necessity, minimizing the number of transfused units, adjusting the transfusion rate, and, when appropriate, administering diuretics as a preventive measure. Although recent platelet transfusion guidelines do not specify an infusion rate, platelet transfusions are generally administered over approximately 60 min per standard adult unit [19].

Considering these principles and the patient’s limited cardiac reserve, platelet transfusions were administered slowly, over 1–2 h per unit. Vital signs, including blood pressure, HR, respiratory rate, and body temperature, were carefully monitored at 15 min intervals to promptly detect any transfusion-related adverse events. CCI was calculated after each transfusion to quantitatively evaluate the platelet response and guide subsequent transfusion decisions. The 1 h CCI was 9.3 × 10^9^/L after transfusion of 6 units, representing an adequate response, and 5.6 × 10^9^/L after transfusion of 2 units, indicating a borderline but non-refractory response. A 1 h CCI ≥ 7.5 × 10^9^/L is generally considered adequate, whereas ≤ 5 × 10^9^/L suggests platelet refractoriness [20]. Antibody screening was negative, and crossmatch-compatible platelets were used, suggesting that the suboptimal increment was attributable to nonimmune factors associated with the underlying MDS rather than immune-mediated refractoriness. Although the CCI is primarily used to assess transfusion efficacy and refractoriness, we also used it serially in this case. The trend in CCI provided a rough estimate of the post-transfusion course and helped guide the transfusion plan toward achieving the target count with minimal volume loading. This approach was consistent with the gradual, response-based strategy described above.

Severe AS remains the dominant intraoperative challenge, despite the optimization of thrombocytopenia. The 2020 ACC/AHA and 2021 ESC/EACTS guidelines do not provide specific numeric targets. Instead, they emphasize the importance of sinus rhythm and prevention of tachycardia, which is considered a major risk factor [4,15,16], especially in patients with coronary artery disease. Case reports have suggested preventing bradycardia (HR < 50 beats/min) and tachycardia (HR > 80–90 beats/min), aiming for a normal or slightly slower HR [3]. Blood pressure management is guided by coronary perfusion pressure rather than absolute values. Some reports have recommended ensuring adequate perfusion by maintaining a DBP of ≥60 mmHg or an MAP of ≥70 mmHg [1,21]. Propofol, a commonly used induction agent, can cause abrupt vasodilation, diastolic hypotension [9,10], which may lead to ischemia, ventricular fibrillation, or cardiac arrest. Vasopressors are often required, and careful titration is advised [9,21]. Etomidate or midazolam may be considered as alternatives [22]. More recently, remimazolam has gained attention for induction and maintenance because of its favorable hemodynamic stability [1,11]. Reduced vasopressor requirements have also been reported with remimazolam [12].

Remimazolam enabled stable hemodynamics without vasopressors. However, transient increases in SBP were observed during periods of surgical stimulation, necessitating remifentanil titration and intermittent beta blockade. These adjustments were performed cautiously to avoid excessive reductions in DBP. These surges were attributed to incomplete suppression of sympathetic responses rather than inadequate hypnosis, as the anesthetic depth was within an adequate range based on the PSI. Although elevated SBP can help maintain coronary perfusion in severe AS, excessive increases may increase afterload, myocardial oxygen consumption, ischemic risk [23], and bleeding tendency. Therefore, SBP should be carefully controlled while maintaining the appropriate DBP and MAP values. The optimal thresholds for these parameters should be individualized according to age, cardiac function, and comorbidities. When blood pressure control is difficult, adjunctive strategies, such as co-administration of low-dose sevoflurane with remimazolam [11,24] or conversion to propofol [25], have been reported to improve hemodynamic stability and sympathetic suppression. Postoperatively, hypertension was mild and transient in the present case. In the PACU, the overall BP remained similar to his pre-induction baseline, although intermittent elevations of SBP above 170 mmHg were observed. At those times, his NRS was 6, and supplemental analgesia was administered, after which the NRS decreased to 4. Despite pain control, intermittent SBP elevations persisted, and β-blockers were cautiously administered to assist with BP control. These findings suggest that the postoperative increase in BP was multifactorial, likely related to pain-induced sympathetic activation in the context of baseline hypertension.

As with all single case reports, the findings should be interpreted with caution. Further studies are needed to refine strategies for managing sympathetic responses and to evaluate the safety and hemodynamic advantages of remimazolam in patients with severe AS.

## 4. Conclusions

This case demonstrates that perioperative management without vasopressor administration is feasible in a patient with severe AS and MDS-related profound thrombocytopenia. In our patient, this was achieved through platelet transfusion titrated to the patient’s response and remimazolam–remifentanil-based total intravenous anesthesia. The key to management was maintaining adequate DBP and MAP while preventing excessive increases in SBP to balance coronary perfusion and afterload. However, intraoperative blood pressure elevations were likely attributable to sympathetic responses to surgical stimuli, which were not completely attenuated by remimazolam. Therefore, adjunctive strategies such as appropriate opioid titration, administration of selective β-blockers, or supplemental use of low-dose volatile anesthetics may be required to achieve optimal hemodynamic control.

## Figures and Tables

**Table 1:** Key preoperative echocardiographic findings.

Parameter	Measured Value	Interpretation
LV ejection fraction	58%	Preserved systolic function
Regional wall motion	None	Normal
LV dimension		
(end-diastolic/end-systolic)	4.5/3.1 cm	Normal
LV volume		
(end-diastolic/end-systolic)	90/38 mL	Normal
LA diameter	4.8 cm	Mild–moderate enlargement
E/E′	19.9	Elevated LV filling pressure
Aortic valve area	0.73 cm^2^	Severe AS
Peak velocity	4.38 m/s	Severe AS
Mean pressure gradient	42.3 mmHg	Severe AS
Maximum pressure gradient	76.8 mmHg	Severe AS

## Data Availability

The data presented in this study are not publicly available due to patient privacy and ethical restrictions. This study was exempt from the Institutional Review Board (IRB No. KNUCH 2025-10-036). De-identified data may be made available from the corresponding author upon reasonable request and with permission from the Institutional Review Board.

## Abbreviations

The following abbreviations are used in this manuscript:

AS	Aortic stenosis
CCI	Corrected count increment
Ce	Effect-site concentration
DBP	Diastolic blood pressure
Hb	Hemoglobin concentration
HLA	Human leukocyte antigen
HR	Heart rate
ICU	Intensive care unit
IV-PCA	Intravenous patient-controlled analgesia
LV	Left ventricular
MAP	Mean arterial pressure
MDS	Myelodysplastic syndrome
NRS	Numerical rating scale
PACU	Post-anesthesia care unit
PLT	Platelet count
PSI	Patient State Index
RBC	Red blood cell
RDP	Random donor platelets
SBP	Systolic blood pressures
SVRI	Systemic vascular resistance index
TACO	Transfusion-associated circulatory overload
TRALI	Transfusion-related acute lung injury
WBC	White blood cell
